# A computer-controlled potentiometric/spectrophotometric titrator

**DOI:** 10.1155/S1463924688000185

**Published:** 1988

**Authors:** John D. Stong

**Affiliations:** Merck Sharp & Dohme Research Laboratories PO Box 2000 Rahway New Jersey 07065 USA

## Abstract

A laboratory computer controlled potentiometric titrator interfaced to a diode array spectrophotometer is described. The titrator consists of widely used, commercially available components; therefore, major attention is given to modes of interconnection and software implementation in data format and system control. Replicate potentiometric titrations of glycines gave a relative standard deviation in titre of 1.035% and a relative standard deviation in pH of 0.745%. Replicate spectrophotometric titrations of bromophenol blue were analysed at three wavelengths to yield pKa= 3.898 ± 0.075 (1.9% rsd).

Methods of data presentation and manipulation are presented.


Journal of Automatic Chemistry, Vol. 10, No. 2 (April–June 1988), pp. 95–100

### A computer-controlled potentiometric/spectrophotometric titrator

**John D. Stong**
*Merck Sharp & Dohme Research Laboratories, PO Box 2000, Rahway, New Jersey 07065, USA*

*A laboratory computer controlled potentiometric titrator interfaced to a diode array spectrophotometer is described. The titrator consists of widely used, commercially available components; therefore, major attention is given to modes of interconnection and software implementation in data format and system control. Replicate potentiometric titrations of glycines gave a relative standard deviation in titre of 1.035% and a relative standard deviation in pH of 0.745%. Replicate spectrophotometric titrations of bromophenol blue were analysed at three wavelengths to yield pKa = 3.898 ± 0.075 (1.9% rsd).*

*Methods of data presentation and manipulation are presented.*

### Introduction

The technique of titrimetry lends itself well to automation. Two general methods for controlling the titration operation have been employed: the older hardware control (i.e. the use of dedicated discrete logic circuits), and more recently, software control (i.e. the use of programmable microprocessors or microcomputers). Both methods have been successfully applied to potentiometric titrations [1–8] and to spectrophotometric/potentiometric titrations [9–13]. Various approaches to the implementation of these techniques are delineated in figure 1.

The two broad categories of control each have advantages and disadvantages. Hardware control generally is less expensive, but severely limits flexibility. Software control utilizing dedicated microprocessors yields much greater flexibility at the expense of greater instrumental complexity. A substantial portion of this complexity can be eliminated by use of a self-contained computer. While such an approach may require the development of interfaces for use between general purpose or personal computers and external equipment, laboratory computers equipped with a wide variety of integrated interfaces and systems drivers callable by high-level programming languages have become widespread.

Additional information and wider scope of operation are available by the incorporation of spectrophotometric analysis of the titrand. This can be realized simply as endpoint determination of a chromophoric titrand at fixed wavelengths [8 and 14], or extensive analysis and data gathering from a complex equilibrium system involving the recording of the entire absorption spectrum [10 and 11]. The latter approach can avail recently devised matrix formalism for manipulating large amounts of data generated by spectrophotometric titrations for the determination of wavelength optima for the analysis of multicomponent systems [14 and 15], for the analysis of multiple equilibria in metal-proton-ligand systems, and in continuous hydrogen-ion titrations of proteins.

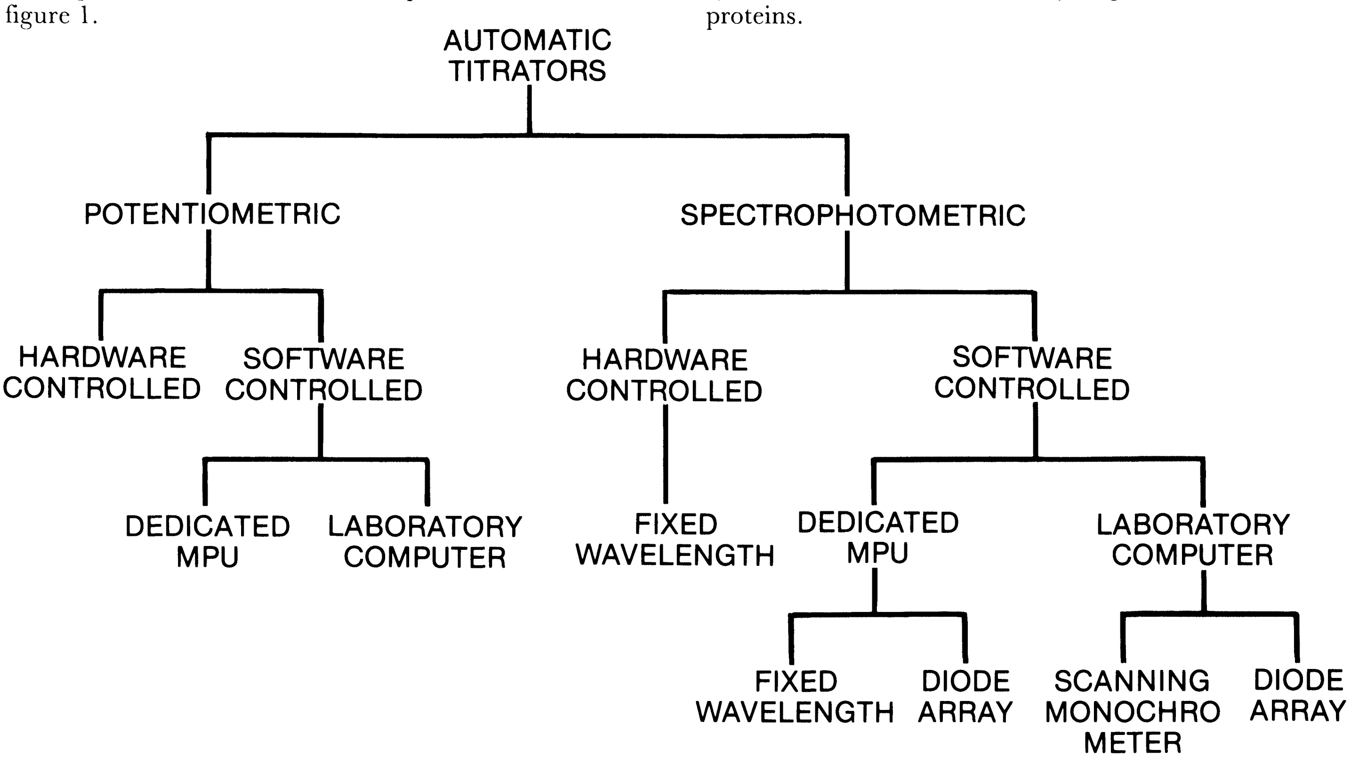

*Figure 1. Delineation of titrator and spectrophotometric titrator designs and implementation.*

Implementation of spectral data gathering has involved the recording of absorbance at fixed wavelengths [8, 12, 13 and 14] and the use of a microprocessor-controlled scanning spectrophotometer [11]. More recently [10], a photodiode array detector was employed in a dedicated microprocessor-controlled titrator which afforded reduced data collection times.

This paper presents a discussion of the construction of a spectrophotometric titration apparatus employing commercially available and widely used equipment. Specifically we describe the use of a Hewlett-Packard 8450A diode array spectrophotometer and a Digital Equipment Corporation MINC-11 laboratory computer, together with other readily available equipment. A minimum of in-house built equipment is used. A detailed discussion of control software, data gathering, storage, manipulation, and presentation is given, as well as general consideration to the use of other computers.

### Experimental

### *Hardware*

*Spectrophotometer:* The spectrophotometer used was the Hewlett-Packard HP8450A diode array spectrometer, with no modifications.

*Computer:* The computer used was the Digital Equipment Corporation MINC-11 laboratory computer, using a PDP 11/03 processor. The unit was equipped with 64K RAM memory, a dual RX02 diskette drive (500 K storage each, double density), parallel 16-bit digital input and output units (DEC MNCDI and MNCDO, respectively), and a four-channel serial RS232 ACS11 interface (DEC DLV11-J). Communication between the HP8450A and the MINC-11 was via the 1200 Baud RS232 serial port. The RXD, TXD, and GND lines were the only ones used, with standard auto-loop back jumpers installed between pins 5 and 8 on the MINC serial unit.

Hard-copy graphics were generated on a Hewlett-Packard HP9872C eight-pen flat bed digital plotter driven by the MINC IEEE-488 interface bus. The programming language used throughout was MINC BASIC V1.2.

*Titration system:* The titration system is comprised of a titrant delivery device and a digital pH meter. The pH meter used was the Orion model 801A digital pH/mV meter. Data from the meter is encoded as parallel BCD. On this particular unit (and all those with serial numbers lower than 7300) the voltage of a logical 1 is +1·5 V, which is too low to ensure recognition by standard TTL circuitry. A simple NPN transistor switch (2N4001) for each BCD bit was employed, which produces an inverted output. The inversion was compensated by setting the data invert switch on the computer's digital input unit.

The titrant delivery system was similar to the one previously described [6]. The Gilmont micrometer burette, 2·5 ml total capacity, is driven directly by a Superior Electric Slo-Syn HS25 stepping motor. The stepping motor is driven by a Slo-Syn model STM103 translator module wired according to the manufacturer's specifications. The module translates serial pulses from the computer into the proper motor coil energization sequence. One motor step (0.9°, or 0.25 μl using a 2.5 ml Gilmont syringe) is produced from each pulse generated by one bit of the computer's digital output unit with the translator set to half-step mode.

The solution being titrated is circulated from a jacketted vessel (about 20 ml total capacity, equipped with an efficient stirrer) through a spectrophotometer flow cell (Fisher) using a Cole-Parmer Masterflex peristaltic pump with viton tubing. The speed of the pump is set to provide complete exchange of the flow cell contents with the titration vessel in 5 s. The burette tip is immersed in the solution continuously. A block diagram of the titrator is shown in figure 2.

### Software

### *Data structure and communication*

*Preliminary consideration:* The system drivers called by MINC BASIC to operate the RS232 interface ports will transmit and receive only serial ASCII, while the HP8450A is capable of sending data in serial eight-bit binary. Such data will always by interpreted as seven-bit ASCII by MINC, thereby losing the most significant of the eight binary bits. It was therefore necessary to instruct the 8450A to send all data as serial ASCII, a much slower process than sending eight-bit binary. ASCII data sent by the HP8450A contain a notation for the wavelength in nm, as, for example, L = 201, followed by the absorbance at that wavelength. The transmission of a 400-point spectrum (200–800 nm) requires 1·8 min at 1200 baud.

*Method of MINC-11 to HP8450A communication:* The HP8450A uses an internal code, referred to as key codes, which consist of numbers (49–109) representing the various keyboard functions (measure, display, plot etc.), and various alphanumeric symbols. To issue a command from MINC-11 to the HP8450A, a command string is constructed by the concatenation of string characters obtained from the operation of the BASIC CHR$ function upon the appropriate HP8450A key codes used in the program.

All keyboard commands to the HP8450A must be preceded by the key code 33 (K$ in table 1) which readies the HP8450A to receive a command string. The command string must then be terminated with an 'execute' command (E$ in table 1) and line-feed/carriage return (LF/CR, T$ in table 1) line terminator. The command string for measuring a spectrum would therefore be constructed as:

Z$ = K$ & M$ & E$ & T$

The string variable Z$ is then passed to the COUT subroutine which transmits the ASCII-coded information on the 1200 baud RS232 lines.

*Structure of records sent by HP8450A:* Table 2 indicates the nominal structure of a single ASCII-coded record received by the MINC-11 from the HP8450A. Details of

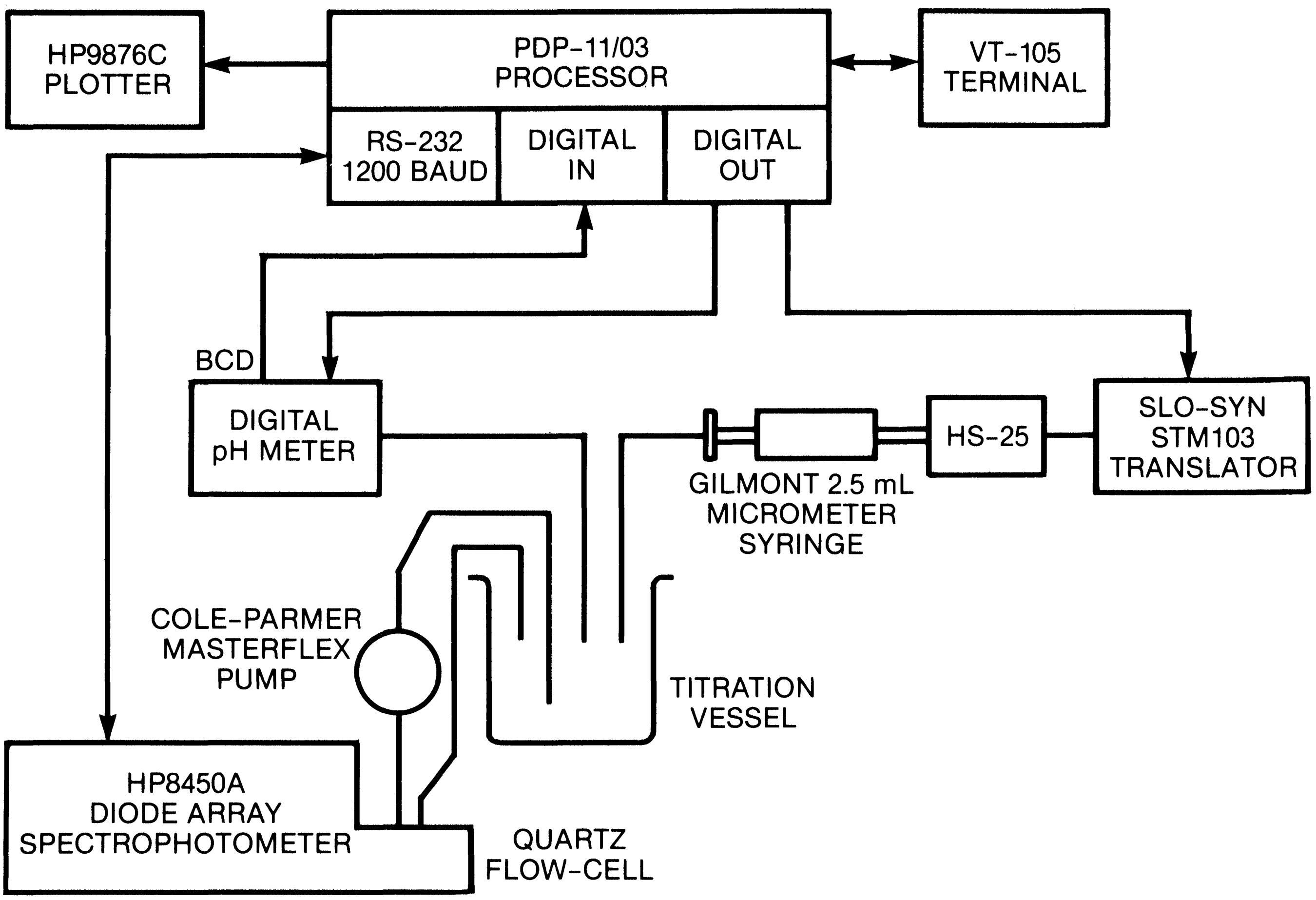

*Figure 2. Block diagram of the spectrophotometric titrator.*

this structure are subject to certain ambiguities, resulting in part from the MINC BASIC CIN (continuous serial input) subroutine, and the HP8450A itself. As indicated in table 2, the first byte contains a line feed character (ASCII 10). This is the result of the 'retrieve' nature of the CIN subroutine. Each record transmitted by the HP8450A is terminated by a carriage return/line feed (CR/LF); CIN waits for the appearance of CR at which point it considers the transmitted record to be complete. The LF character arriving after the CR terminator is picked up (retrieved) by CIN with the next record. Hence, records arriving after the first one will begin with LF. It was found that the very first record transmitted after measuring a spectrum and sending it to the computer will begin with the characters 0 (zero, ASCII 48), or occasionally with a LF, 0 (ASCII 10, ASCII 48) as indicated in table 2, alternate structure. It is therefore necessary to execute software which will examine each

*Table 1. Key-code ASCII equivalents.*

| | |
|---|---|
| K$ = CHR$ (33) | : Keyboard Command Follows |
| E$ = CHR$ (94) | : Execute |
| T$ = CHR$ (13) & CHR$ (10) | : CR/LF, Line Terminator |
| R$ = CHR$ (125) | : Remote |
| L$ = CHR$ (124) | : Local |
| EO$ = CHR$ (55) | : Erase |
| M$ = CHR$ (48) | : Measure |
| TI$ = CHR$ (58) | : Time |
| B$ = CHR$ (57) | : Balance |
| W$ = CHR$ (66) | : Wavelength |
| A$ = CHR$ (89) | : Absorbance |
| TO$ = CHR$ (90) | : To |
| D$ = CHR$ (80) | : Device |
| P2$ = CHR$ (72) | : Plot |
| Y$ = CHRR (50) | : Y-Scale |
| U$ = CHR$ (101) | : UV |
| V$ = CHR$ (109) | : Visible |
| D4$ = D$ & CHR$ (85) & CHR$ (83) | : Device 31, Computer, ASCII |

*Table 2. Data structure received from HP8450A by the CIN subroutine.*

| Record | Content | |
|---|---|---|
| 1 | Line-feed | (ASCII 10) |
| 2 | L | (ASCII 76) |
| 3 | Space | (ASCII 32) |
| 4,5,6 | Wavelength value | |
| 7 | = | (ASCII 61) |
| 8 | Space | (ASCII 32) |
| 9–14 | Absorbance value[1] | |
| Alternate #1 | | |
| 1 | 0 | (ASCII 48) |
| Alternate #2 | | |
| 1 | Line-feed | (ASCII 10) |
| 2 | 0 | (ASCII 48) |

1. Record 9 will contain an asterisk (ASCII 42) if the absorbance value is greater than 4·00.

record and extract the absorbance from amidst these ambiguities and contingencies. Such software has been written and is available.

*Reception and storage of data by the MINC-11 computer:* Data sent to the MINC-11 computer by the HP8450A are stored initially in a string array. The array is dimensioned for the maximum number of absorbance values that can be contained in a spectrum recorded by the HP8450A (400) plus two extra elements to accommodate the spurious inclusion of line feed and zero characters, as noted above. Data are received by the MINC in a loop containing only a call to the CIN subroutine, which is executed two more times than the expected number of transmitted absorbance values. The argument passed to the CIN subroutine includes the string array element in which the current absorbance value is to be stored. Each element of the string array is defined as being of variable length, so that CIN waits for a carriage return (ASCII 13) at which point the data record is regarded as complete and control is returned to the calling program. A time-out interval of 2s (the time CIN waits for the appearance of a carriage return before default exiting) was found to be the optimum value.

### Titration control

*Initialization of the spectrometer*

The communication and data transfer between the HP8450A and the MINC-11 require that the data byte structures and baud rates of both machines be identical. The CIN subroutine affords only one totally reliable rate, 1200 baud. Additionally, no provision is made by the computer to allow alteration of the serial data byte structure. The HP8450A, however, allows modification of its data byte structure with the 'device 43' command entered from the spectrometer keyboard as Device 43, 7, 4. This sets the baud rate to 1200, and the data byte to one stop bit and no parity, in conformity with the MINC-11 requirements. The computer can now place the HP8450A in 'remote' by issuing the R$ string (see table 1) embedded in the command string:

Z$ = K$ & R$ & T$

where Z$ becomes the argument to the COUT subroutine.

At this point the deuterium and tungsten lamps are turned on by the computer and allowed to warm up for 25 min. After warm-up, another 'lamp on' command is issued to allow correct diode amplifier gain settings to be established. A balance measurement is then made by the computer (with or without cells in sample and reference compartments, at the operator's discretion).

*Setting spectral parameters*

It is often desirable to alter the default spectral width parameters (200–800 nm). The time required for transmission of a spectrum increases with the width of the spectrum, and attention is often confined to a particular spectral region. It is also sometimes necessary to set fixed upper and lower absorbance limits. These limits are entered into the computer as character strings and must be translated into the correct key-code command strings. The character strings are stored as elements of a string array. Each element of the array is 'decomposed' by the SEG$ operator, which is set to return a single character from the argument string, in order from first to last. Each single character substring is then translated into the corresponding key-code by reference to a translation table. The individual component key-code values are stored sequentially in a numeric string. Thus, the first three elements of this array contain the key-code equivalents for each of the three digits of the lower wavelength limit. The next three elements contain the upper limit, the next three the lower absorbance limit, and the last three the upper absorbance limit. Each key-code number is for a given value converted to a character (with the CHR$ operator) and concatenated to form the key-code character strings of the lower and upper wavelengths for a spectrum. The command string to set the HP8450A to these values would be:

Z$ = K$ & W$ & LO$ & TO$ & LI$ & E$ & T$

where (consulting table 1)

K$ = key-code for keyboard command.
W$ = key-code for wavelength.
LO$ = code for lower wavelength.
LI$ = code for upper wavelength.
TO$ = key-code for 'to'.
E$ = key-code for execute.
T$ = line terminator.

### Results and discussion

The major purpose of the titrator described here was to obtain a set of absorption spectra corresponding to a series of pH or other potentiometric measurements in machine-readable form in the quickest, simplest and most reliable manner available to us. The accuracy is

*Table 3. pKa calculated from three separate titrations of bromophenol blue.[1]*

| File/λ(nm) | BPB04 | BPB03 | BPB02 | |
|---|---|---|---|---|
| 309 | 3·727 | 3·810 | 3·953 | 3·830 ± 0·114 (3·0%) |
| 588 | 3·848 | 3·914 | 3·973 | 3·912 ± 0·063 (1·6%) |
| 436 | 3·927 | 3·914 | 4·014 | 3·952 ± 0·054 (1·4%) |
| | 3·934 ± 0·101 (2·6%) | 3·879 ± 0·060 (1·5%) | 3·980 ± 0·031 (0·8%) | |

[1]λ(nm) = wavelength at which calculation was performed; numbers in last row and column are mean ± std dev (rsd).

dependent primarily upon the potentiometric measurement system, the titrant delivery system, and the spectrophotometer. The computer does not materially affect the accuracy of the system, except to the extent that it determines the number of steps taken by the stepping motor. Replicate titrations ($N = 3$) of glycine at a concentration of $7 \times 10^{-3}$M plus 0·48–0·50 $\times 10^{-3}$M HCl provide information on the repeatability of the titrant delivery system and the pH measurement system. The average titre for the first end-point was 499·0 ± 1·6 μl (rsd = 0·3%), and the average titre for the second end-point was 1211·6 ± 13·0 ml (rsd = 1·1%). The average pH at the first end-point was 6·023 ± 90·127 (rsd = 2·1%) and at the second point it was 10·969 ± 0·054 (0·5%). The end-points were determined by locating the maximum value of the forward difference ratio pH/V. The identical titrations were also compared on a point-by-point basis, yielding a set of 93 averages ($N = 3$) and standard deviations. The average relative standard deviation for 93 values of titre and pH were then computed, giving rsd titre = 1·035% and rsd pH = 0·745%.

Replicate spectrophotometric titrations ($N = 3$) of bromophenol blue were performed. Each titration was analysed at 588 nm, 436 nm, and 309 nm as the

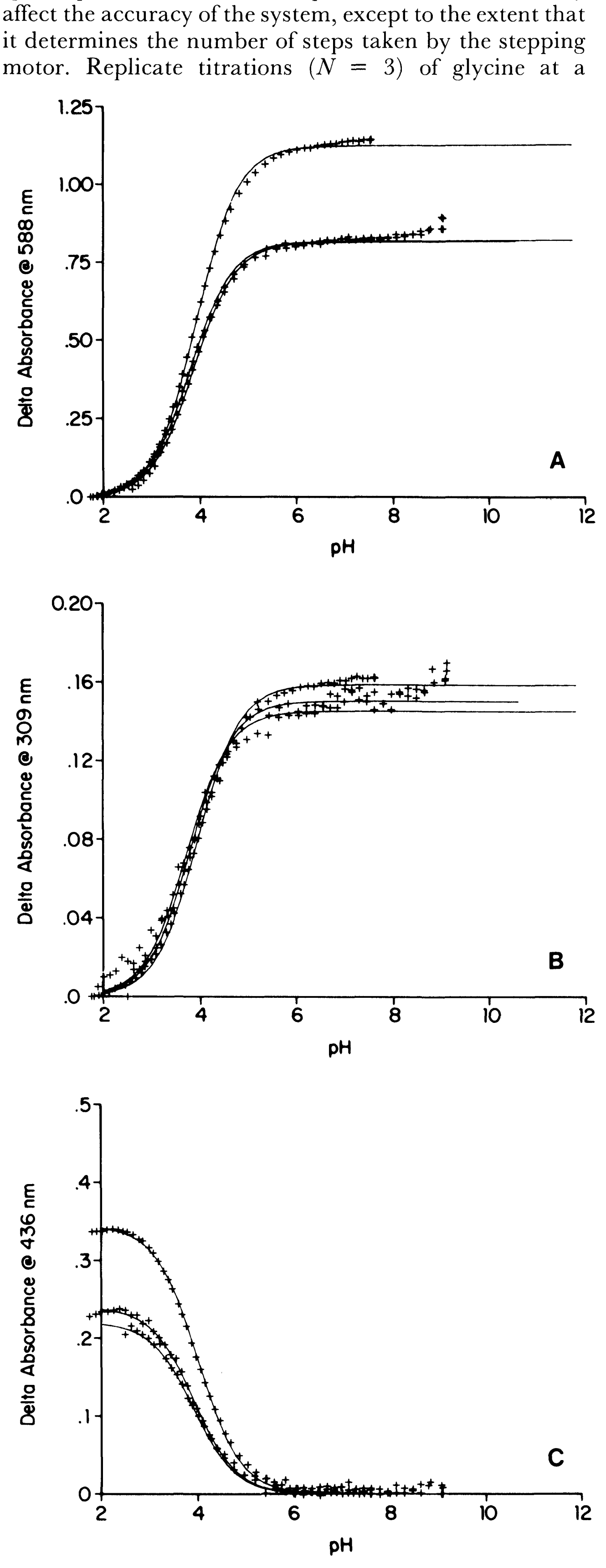

*Figure 3. Data (+) and calculated best fit for the titrations of bromophenol blue at A: 588 nm; B: 309 nm; C: 436 nm.*

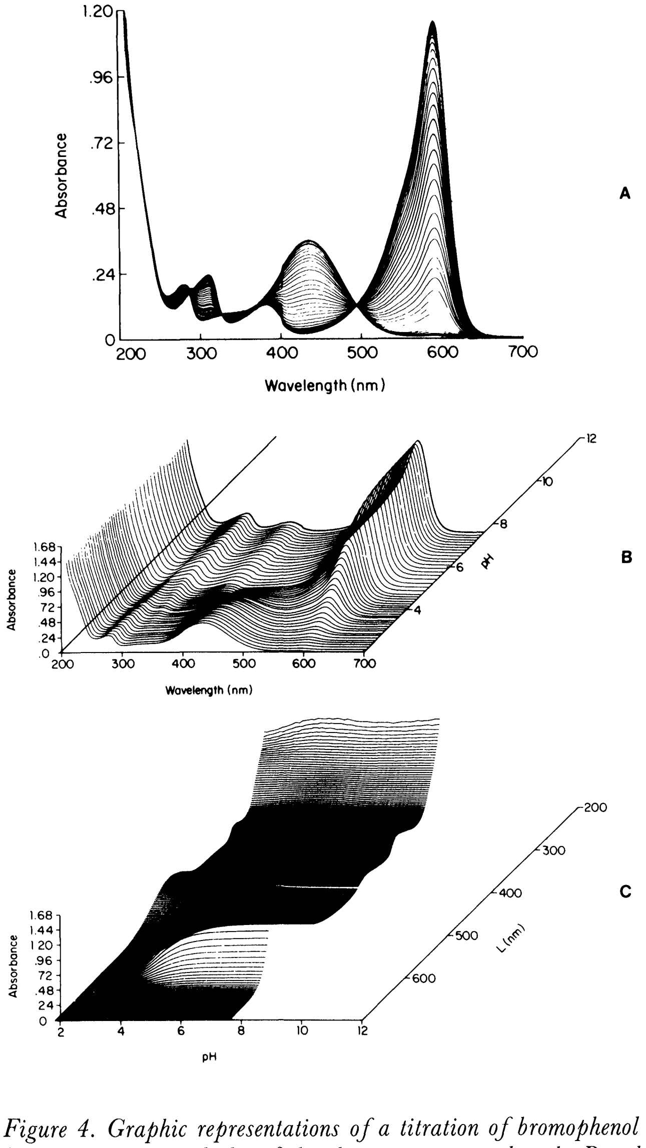

*Figure 4. Graphic representations of a titration of bromophenol blue. A: conventional plot of absorbance versus wavelength; B and C: rotation of the data in A, showing each axis (absorbance, wavelength, and pH) in three dimensions.*

dissociation of a monoprotic acid according to the equation

$$\Delta A = \frac{K_a}{h + K_a} \Delta A_{\text{max}}$$

at = 588 and 309 nm, and

$$\Delta A = \left(1 - \frac{K_a}{h + K_a}\right) \Delta A_{\text{max}}$$

at = 436 nm, and where
$\Delta A$ = change in absorbance
$\Delta A_{\text{max}}$ = maximum (total change in absorbance)
$K_a$ = dissociation constant
$h$ = $10^{-\text{pH}}$, where pH = pH meter reading.

A computer program varied the values of $K_a$ and $A_{\text{max}}$ in order to minimize the error square sum

$$(\Delta A_{\text{exp}} - \Delta A_{\text{calc}})^2.$$

The results of these calculations are shown in table 3, which gives pKa values as a function of experiment and wavelength. The average value for all experiments at a particular wavelength was 3·898 ± 0·062 (1·6%) and the average value for all experiments at all wavelengths was 3·898 ± 0·075 (1·9%). The data and simulated curves are shown in figure 3.

A particular advantage of machine-readable data is the flexibility imparted to data manipulation, analysis, and presentation. As an example, a single pH titration of bromophenol blue is presented in figure 4 in conventional format, and in two three-dimensional formats. The latter produce a titration surface, which in the case of more complex systems would be capable of revealing additional information which might be obscured in more conventional representations.

Aside from using other source languages, the major difference expected in using other computers would be in the RS232 drivers. A more flexible system which allows reception of eight-bit binary would be more efficient in terms of transmission time, but more rigid in that the entire spectrum (1664 bytes) is sent in binary mode, regardless of the requested spectral width parameters. Transmission by the computer in RS232 would be facilitated by other drivers which allow inclusion of the actual output data in the subroutine argument without need for the generation of cumbersome command strings, as well as the mode in which it is to be sent. The actual transmission format as presented here is completely general, however.

### References

1. Hunter, T. W., Sinnamon, J. T. and Heiftje, G. M., *Analytical Chemistry,* **47** (1975), 497–502.
2. Velinov, G., Todorov, N. and Karamphilov, S., *Talanta,* **30** (1983), 678–691.
3. Fruh, P. U., Meier, L., Rutishauser, H. and Siroky, O., *Analytica Chemica Acta,* **95** (1977), 97–106.
4. Chipperfield, J. R., Roscoe, R. M. and Webster, D. E., *Analytical Proceedings,* **20** (1983), 127–130.
5. Frazer, J. W., Kray, A. M., Selig, W. and Lim, R., *Analytical Chemistry,* **47** (1975), 869–875.
6. Avdeef, A. and Bucher, J. J., *Analytical Chemistry,* **50** (1978), 2137–2142.
7. Wu, A. H. B. and Malmstadt, H. V., *Analytical Chemistry,* **50** (1978), 2090–2096.
8. Pehrsson, L. and Ingman, F., *Talanta,* **24** (1977), 79–85.
9. Wu, A. H. B., Rotunno, T. and Malmstadt, H. V., *International Laboratory,* **12** (1982), 16–24.
10. Hanisch, G., Kaden, T. A. and Zuberbuhler, A. D., *Talanta,* **26** (1979), 563–567.
11. Lindberg, A. O., *Analytica Chimica Acta,* **96** (1978), 319–325.
12. Jongkind, W., Eelderink, G. H. B., Verbruggen, H. B., van Oort, W. J. and Gripink, B., *Z. Analytical Chemistry,* **286** (1977), 72–75.
13. Pehrsson, L. and Ingman, F., *Talanta,* **24** (1977), 87–90.
14. Frans, S. D. and Harris, J. M., *Analytical Chemistry,* **57** (1985), 2680–2684.
15. Brown, C. W., Lynch, P. F., Obremski, R. J. and Lavery, D. S., *Analytical Chemistry,* **54** (1982), 1472–1479.

### LABORATORY INFORMATION MANAGEMENT SYSTEMS

A meeting of the East Anglia Region of the Analytical Division of the Royal Society of Chemistry, will be held on 5 May 1988 at Smith Kline & French Labs Ltd, Mundells, Welwyn Garden City, UK. The programme is as follows:

The requirement specifications for LIMS, *by Dr B. McDowall (SK&F Research Ltd, Welwyn).*

LIMS (Beckman) in the pesticide residue laboratory, *by P. Snowdon (Schering, Saffron Walden).*

Current and future impact of LIMS (Perkin-Elmer) in quality control, *by Dr A. Wayland (Roche, Welwyn Garden City).*

HP LABSAM in an integrated system, *by I. Smith (Glaxo, Ware).*

LIMS (Beckman) for the end user, *by A. D. Henderson (SK&F Laboratories Ltd, Welwyn Garden City).*

*There is a registration fee for this meeting. Further details can be obtained from the Analytical Division, Royal Society of Chemistry, Burlington House, London W1V 0BN.*

